# A systematic review of interventions in the early course of bipolar disorder I or II: a report of the International Society for Bipolar Disorders Taskforce on early intervention

**DOI:** 10.1186/s40345-022-00275-3

**Published:** 2023-01-03

**Authors:** A. Ratheesh, D. Hett, J. Ramain, E. Wong, L. Berk, P. Conus, M. A. Fristad, T. Goldstein, M. Hillegers, S. Jauhar, L. V. Kessing, D. J. Miklowitz, G. Murray, J. Scott, M. Tohen, L. N. Yatham, A. H. Young, M. Berk, S. Marwaha

**Affiliations:** 1 Orygen, 35 Poplar Road, Parkville, VIC Australia; 2 Centre for Youth Mental Health, The University of Melbourne, Melbourne, Australia; 3 Institute for Mental Health, University of Birmingham, Birmingham, UK; 4 Birmingham and Solihull Mental Health NHS Trust, Birmingham, UK; 5 TIPP Program, Service of General Psychiatry, Department of Psychiatry, Lausanne University Hospital and Lausanne University, Lausanne, Switzerland; 6 IMPACT-The Institute for Mental and Physical Health and Clinical Translation, School of Medicine, Barwon Health, Deakin University, Geelong, Australia; 7 Nationwide Children’s Hospital, The Ohio State University, Columbus, USA; 8 Department of Psychiatry, University of Pittsburgh, Pittsburgh, USA; 9 Department of Child and Adolescent Psychiatry/Psychology, Erasmus Medical Centre, Rotterdam, The Netherlands; 10 Department of Psychological Medicine, Institute of Psychiatry, Psychology and Neuroscience, King’s College London, London, UK; 11 South London and Maudsley NHS Foundation Trust, Bethlem Royal Hospital, Monks Orchard Road, Beckenham, Kent, BR3 3BX UK; 12 Copenhagen Affective Disorder Research Center (CADIC), Psychiatric Center Copenhagen, Copenhagen, Denmark; 13 Department of Clinical Medicine, University of Copenhagen, Copenhagen, Denmark; 14 Semel Institute for Neuroscience and Human Behavior, Los Angeles School of Medicine, University of California, Los Angeles, USA; 15 Centre for Mental Health, Swinburne University of Technology, Melbourne, Australia; 16 Institute of Neuroscience, Newcastle University, Newcastle Upon Tyne, UK; 17 Department of Psychiatry and Behavioral Sciences, University of New Mexico, Albuquerque, NM USA; 18 Department of Psychiatry, University of British Columbia, Vancouver, Canada

**Keywords:** Bipolar disorder, Early intervention, Course, Lithium, Mood stabilisers, Antipsychotics, CBT, Psychoeducation, Mania, Depression, Remission, Recurrence, Systematic review

## Abstract

**Background:**

Given the likelihood of progressive illness in bipolar disorder (BD), it is important to understand the benefits and risks of interventions administered early in illness course. We conducted a systematic review of the effectiveness of interventions in the early course of BD I or II.

**Methods:**

We completed a systematic search on MEDLINE, PsycINFO, EMBASE, the Cochrane Central Register of Controlled Trials, CINAHL and Google Scholar from 1/1/1979 till 14/9/2022. We included controlled trials examining intervention effects on symptomatic, course, functional and tolerability outcomes of patients in the ‘early course’ of BD I or II. We classified patients to be in early course if they (a) were seeking help for the first time for a manic episode, (b) had a lifetime history of up to 3 manic episodes, or (c) had up to 6 lifetime mood episodes. Evidence quality was assessed using the GRADE approach.

**Results:**

From 4135 unique publications we included 25 reports representing 2212 participants in 16 randomized studies, and 17,714 participants from nine non-randomized studies. Available evidence suggested that in early illness course, lithium use was associated with lower recurrence risk compared with other mood stabilizers. Mood stabilizers were also associated with better global functioning, compared with the use of antipsychotics in the medium term. While summative findings regarding psychological therapies were limited by heterogeneity, family-focused and cognitive-behavioral interventions were associated with reduced recurrence risk or improved symptomatic outcomes. There was some evidence that the same pharmacological interventions were more efficacious in preventing recurrences when utilized in earlier rather than later illness course.

**Conclusions and recommendations:**

While there are promising initial findings, there is a need for more adequately powered trials to examine the efficacy and tolerability of interventions in youth and adults in early illness course. Specifically, there is a compelling need to compare the relative benefits of lithium with other pharmacological agents in preventing recurrences. In addition to symptomatic outcomes, there should be a greater focus on functional impact and tolerability. Effective pharmacological and psychological interventions should be offered to those in early course of BD, balancing potential risks using shared decision-making approaches.

**Supplementary Information:**

The online version contains supplementary material available at 10.1186/s40345-022-00275-3.

## Background

Bipolar disorder (BD) is a recurrent and severe mood disorder contributing to global disability (Whiteford et al. 2013) likely due to its early onset (Geoffroy et al. 2013), relapsing and remitting course, and impacts on education, employment and cohabitation (Marwaha et al. 2013; Conus et al. 2014; Sletved et al. 2021). It has been argued that earlier use of evidence-based treatments may have a protective effect and could mitigate disability associated with the disorder (Vieta et al. 2018; Jauhar et al. 2019). Early intervention can refer to populations who are at-risk for the disorder before full diagnostic criteria are met (Kupka et al. 2021). While a recent systematic review investigated the evidence for early interventions in cohorts at high risk of developing BD (Saraf et al. 2021), there is a compelling need to examine the role of interventions in early illness course after onset of fully syndromal bipolar disorder. This is because one cannot assume the window for early intervention has closed for all persons once BD has been diagnosed, or after the first episode of mania. In fact, many authors concur that targeted treatments should generally be offered only when BD has been diagnosed (Malhi et al. 2017), and this may be a balanced approach to optimize recovery. The early post-onset course of BD could therefore be similar to the ‘critical period’ for secondary prevention described in early psychosis (Birchwood et al. 1998).

Identifying risks and benefits of interventions early in the course of diagnosable BD can help identify secondary prevention approaches (Haggerty and Mrazek 1994) including that of comorbid conditions and inform clinical practice guidelines. While several excellent guidelines are available for the care of persons with BD (Goodwin et al. 2016; Grunze et al. 2009, 2010, 2013; Yatham et al. 2018; Malhi et al. 2021), these guidelines do not distinguish recommendations for those in the earlier vs. later course of illness. Interventions for children and adolescents may receive separate attention (Goodwin et al. 2016; Yatham et al. 2018; Goldstein et al. 2017) but a substantial proportion of patients with BD have an onset in adulthood (Geoffroy et al. 2013; Post et al. 2008) and a minority even in late life (Tohen et al. 1994). Thus, there is a need to examine the impact of interventions early in illness course more broadly, not just early in chronological age. There has not been a systematic evaluation of the effectiveness of interventions among those diagnosed within a few episodes of onset of BD I or II across age ranges. We conducted a systematic review of the clinical effectiveness of interventions among those in the early course of BD I or II. Our primary objective was to describe the evidence for interventions among those with relatively few episodes after illness onset. The secondary objective was to examine whether interventions led to different outcomes in early and later illness course. If interventions had greater effectiveness in the early illness course, this could support the hypothesized critical period for early intervention.

## Materials and methods

The study followed a peer-reviewed protocol (registered on the Prospero website: CRD42020195956) and adheres to the Preferred Reporting Items for Systematic Reviews and Meta-Analyses (PRISMA, 2020 (Page et al. 2021), Additional file 1: Table S2) and Synthesis Without Meta-Analysis (SWIM (Campbell et al. 2020)) guidelines.

### Eligibility criteria

We used the following inclusion criteria:

#### Population

The majority of participants in the study should be diagnosed with BD I or BD II (based on DSM III-R, IV or 5, ICD-9, 10 or 11) in any polarity or phase of illness, with no age restrictions.

#### Illness course

The study sample must wholly or partially be comprised of participants in the early illness course. There is no international consensus definition of the early course of BD. As such, our expert group agreed a priori that we would examine interventions offered to individuals who presented with the following illness patterns: (i) first treatment seeking episode of mania or (ii) first three manic episodes lifetime or (iii) not more than six mood episodes lifetime. First treatment-seeking episode of mania was operationalised as first hospitalisation for mania. Although somewhat disparate, these definitions were arrived at via consensus among taskforce members, based on existing literature. Multiple definitions were utilized in order to increase the scope of the review and maximise usefulness of conclusions to researcher and practicing clinicians. In combining the three definitions, we considered that those seeking treatment for their first episode of mania often include participants with several prior depressive or hypomanic episodes (Berk et al. 2007). Similarly, our definition allowed us to include BD types I and II with the first two referring to BD type I and the third to BD II. While our definition initially referred to ‘early stage’ BD in our published protocol, we noted the lack of clarity regarding the definition of early illness stages in the ISBD Staging Nomenclature Taskforce (Kupka et al. 2021). Therefore, we clarified our focus to be early illness course to limit ambiguity. We did not include studies that defined early illness course using time elapsed from diagnosis or illness onset (e.g., first 2 years of illness), given difficulties in ascertaining illness onset and the risk of making the review population more heterogeneous.

#### Intervention

Any psychopharmacological intervention (e.g., mood stabilizers, antipsychotics, antidepressants), psychological intervention (e.g., cognitive-behavioral therapy [CBT], psychoeducation, family therapy), neurostimulation, nutraceutical agent or a combination. We defined ‘mood stabilizers’ to include lithium and anticonvulsants but not antipsychotics for this study.

#### Comparisons

Included either (i) between group comparisons within early course of illness (e.g., medications versus placebo or active comparator or psychological interventions versus waiting list or control condition or another psychological interventions) or (ii) comparison of efficacy of the same intervention offered to those in the early course of BD compared with other illness course.

#### Outcomes

Studies were included if they reported ≥ 1 of the following: (i) symptomatic change, remission or recovery (manic symptoms, depressive symptoms, clinical global impression scores); (ii) categorical or continuous estimates of relapse, recurrence or rehospitalizations; (iii) functional status; or (iv) tolerability of intervention.

#### Study design

Randomised or non-randomised intervention study with a comparison arm.

#### Exclusion criteria

Articles not published in English language or published before 1979 (publication date of ICD-9), and studies that focused on patients with Other Specified Bipolar and Related Disorders (OSBARD) or BD Not Otherwise Specified (BD NOS), or prodrome, due to lack of operational clarity on the lower threshold for these conditions as outlined above. We excluded case series or individual case reports relating to interventions.

### Search strategy

#### Data sources

MEDLINE, PsycINFO, EMBASE, the Cochrane Central Register of Controlled Trials, CINAHL and Google Scholar until 14/9/2022.

#### Search terms

Search terms included MeSH terms and were arranged in groups; Group 1 for population: bipolar or "bipolar depression" or "manic depress*" or mania or manic or hypomania or hypomanic, AND Group 2 for stage: (Early or first) and (stage* or episode* or course) OR stage* or staging or "number of episodes" or “illness course” or episode* or first episode mania or first episode hypomania or first contact or first psychiatric contact AND Group 3 for interventions: intervention* or treatment* or therapy or medication* or neurostimulation or antipsychotic* or anticonvulsant* or valpro* or divalpro* or lithium or lamotrigine or mood stabilizer* or mood stabiliser* or psychological or cognitive or behavioural or behavioral or psychoanaly* or supportive or interpersonal or social rhythm or psychoeducation or neutraceutical or nutrition AND Group 4 for study design: trial or controlled study or random* control* trial* or RCT* or observational or naturalistic or cohort or prospective or longitudinal or registry or register* AND Group 5 for outcomes: response or symptom* or relapse or recurrence or hospital* or function* or quality of life or recovery or side effects or tolerab* or time to discontinuation.

In addition, studies that were known to study investigators, or those identified from reference or ancestry searching were considered for inclusion. We contacted investigators in the field to determine if they had other relevant data available (e.g., other publications or ongoing research) and contacted authors to obtain additional information and/or to obtain separate data regarding those in early illness course.

### Study selection

Articles were initially screened independently based on title and abstract by two reviewers (EW and DH), with full text obtained for those fulfilling eligibility criteria. The initial screening and coding of eligibility was completed independently without direct collaboration between EW and DH to reduce bias. Any uncertainties regarding eligibility were then resolved by a third reviewer (AR). We also contacted authors to get further details in cases where it was unclear whether their article met inclusion criteria for this review.

### Data extraction

Data were extracted by EW, AR and JR using a customized data extraction form, which was piloted before commencing data extraction. Pilot data extraction was completed by EW for five RCTs and five non-randomised studies, under the supervision of another reviewer (AR). At this stage, we aimed to examine the completeness of data available and agree on definitions of outcomes selected for extraction.

### Quality assessment

The Cochrane assessment of Risk of Bias 2 (Higgins et al. 2011) and the companion tool focused on Non-Randomised study designs (ROBINS) (Sterne et al. 2016) were used to assess quality of randomized controlled trials and observational studies, respectively. Ratings were performed independently by AR and DH, and discrepant ratings resolved in consensus with SM.

### Qualitative synthesis

We described findings relating to the interventions identified, including randomized vs non-randomized comparisons, further grouped into comparisons (a) within early illness course, and (b) across early and later course of illness. In comparing early vs later illness course, studies could compare subgroups by separating first episode manic participants from multi-episode participants or by using a cut-off of either (a) lifetime mood episodes from 1 through 6, or (b), lifetime manic episodes from 1 through 3. For categorical outcomes such as remission, response, and adverse events we reported adjusted or unadjusted odds ratios. For survival related measures such as time to recurrence or relapse, we reported adjusted or unadjusted hazard ratios when available. Mean differences were reported for continuous measures to enable interpretability of the measure reported. When effect size differences were not reported, these were estimated (if possible). Studies with lower or higher risk of bias are highlighted in text, while the remaining studies with intermediate or moderate risk of bias are described only in tables. For summation of evidence, we used vote counting based on direction of effect, and investigated heterogeneity when there were two or more comparisons using the same or similar interventions for each outcome, in a similar time period. Studies were grouped at the level of the specific intervention when enough studies were available for the same comparison or at a meaningful category of intervention (e.g., pharmacotherapy, mood stabiliser) when there were relatively few studies at the individual intervention level. Certainty of evidence was described using GRADE criteria (Guyatt et al. 2008) when two or more such comparisons were available. This was based on SWIM recommendations and represents a change from our a priori data synthesis plan.

## Results

### Selected articles

**Fig. 1:**
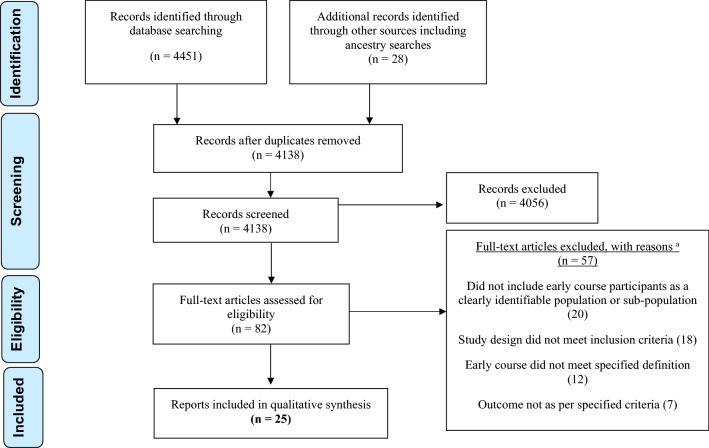
Flow diagram

Our search strategy yielded 4451 publications; an additional 28 articles were considered for inclusion from other sources. After removing duplicates and applying selection criteria, 82 full-text articles were assessed for eligibility from which 25 were included. Among these, three papers were included based on additional data provided by the authors (Hafeman et al. 2020; Inder et al. 2015; Miklowitz et al. 2003). The PRISMA flowchart (Fig. 1) illustrates the number of papers included and excluded at each step.

Table 1 describes the design and main findings of included studies. We included 16 RCTs and nine non-randomized studies. These represented 2212 unique individuals in randomised comparisons and 17,714 participants from non-randomised intervention study comparisons. The most common definition of early course was inclusion of those in their first treated episode of mania (9 studies), followed by studies that included participants (or subgroups of participants) with 3, 5 or 6 lifetime mood episodes.

**Table 1:** Details of included studies

Authors	Study design/comparison/population	Sample size	Age, yrs (M ± SD or median, range)	% Female	Outcome/s measured	Main findings	Comments
I. Interventions in early course of illness							
*Randomised controlled trials*							
Berk (2017)	RCT comparing maintenance treatment with quetiapine or lithium in patients with recently stabilised first-episode mania with psychotic features (15–25 yrs)	61	21.3 ± 2.3	22%	Symptomatic change, Global Illness Severity, Functioning, Quality of life	Continuation treatment with lithium rather than quetiapine resulted in more favorable mean levels of symptoms and functioning over a 1-year period	Planned secondary analysis and post-hoc analyses
Conus et al. (2015)	RCT comparing the efficacy of chlorpromazine vs olanzapine in patients with first episode of psychotic mania (15–25 yrs)	83	21.5 ± 2.9	32.4%	Efficacy (response on mania and depression scales)	Olanzapine was similar to chlorpromazine in achieving remission from manic symptoms	A priori primary hypothesis with adequate power
Detke et al. (2015)	RCT of Olanzapine-Fluoxetine (OFC) combination vs placebo in adolescents (10–17 yrs) with BD Depression	291	14.7 ± 2.3	49%	Depressive symptoms, adverse events	OFC combination was superior to placebo in adolescents in treating acute depression in the early course of BD	A priori primary hypothesis with adequate power
Kessing (2013) Kessing et al. (2014a)	RCT comparing treatment in a specialized mood clinic vs standard care in early course of BD	158	Median 35.6 years, quartiles 27.7, 47.1	54.4%	Readmission to hospital, Treatment satisfaction	Treatment in a specialized mood disorder clinic significantly reduced time to readmissions and improved treatment satisfaction compared with standard care. Those aged 18–25 years had a trend towards more pronounced benefit in specialized treatment increasing time to recurrence (p = 0.05)	A priori primary hypothesis with adequate power
Miklowitz et al. (2003)	RCT comparing Family Focused Psychoeducation (FFT) + pharmacotherapy or crisis management + pharmacotherapy among adults. A majority of the sample was in an early course of illness with ≤ 6 prior episodes	101	35.6 ± 10.2	63.3%	Time to relapse and symptom severity	A majority of participants were in early illness course. Participants receiving FFT had fewer relapses (35% vs 54%) and a longer time to relapse (74 weeks vs 53 weeks). FFT was also associated with better symptomatic control and improved medication adherence	A priori primary hypothesis with adequate power
Miklowitz et al. (2008)	RCT of FFT tailored for Adolescents (FFT-A) compared with Enhanced Care (EC) among adolescents, with both groups receiving pharmacotherapy. FFT was delivered over 21 sessions	58	14.5 ± 1.6	56.9%	Time to recovery and recurrence	There were no group differences on overall time to recovery and recurrence. FFT-A was associated with quicker recovery from depressive episodes and spent fewer weeks in depressive episodes	A priori primary hypothesis with adequate power
Miklowitz et al. (2014)	RCT of FFT-A among adolescents with BD I or II, compared with 3 session family psychoeducation, with both groups receiving pharmacotherapy	145	15.6 ± 1.4	54.5%	Time to recovery and recurrence	There were no group differences in time to recovery and recurrence. Those in FFT-A had greater improvements in manic symptoms particularly between 12 and 24 months	A priori primary hypothesis with adequate power
Patino et al. (2021)	RCT comparing lithium and quetiapine in acute treatment of mania among youth aged 10–17 years, with 85% of the sample having had fewer than 3 prior episodes	107	14.2–15.1 ± 1.9–2.1	57–63%	Response with respect to manic symptoms	Participants treated with quetiapine were more likely to respond (72.4%) than those treated with lithium (49%, p = 0.012). Remission rates did not differ between groups, and symptoms or CGI severity did not differ at end-point	A priori primary outcome was symptom change, response was secondary
Perry et al. (1999)	RCT examining efficacy of education regarding early warning signs of a relapse (psychoeducation). The sample had 5–6 median lifetime bipolar episodes indicating that a majority of the sample was in early illness course	69	44–45 (SD 11–13)	68–69%	Manic or depressive relapses	Time to first manic relapse was significantly longer with respect to manic episodes (25%, 65 wks in experimental group and 17 wks in control group). No significant differences noted for depressive relapses. Total number of manic episodes were fewer (2 vs 11 respectively)	A priori primary hypothesis with adequate power. Likely that predominantly hospitalised episodes were included in count of prior episodes
Strakowski et al. (2016)	Pseudo-Randomized Controlled Trial examining effect of quetiapine or lithium treatment for a first manic episode on brain activation changes. Participants could deviate from assigned intervention	42	18 ± 5 (13–25 years)	60%	Response on mania scales reported as a secondary outcome	Lithium numerically associated with higher response rate (58%) than quetiapine (43%), but this was not statistically significant	Incidental report on outcomes, likely insufficient power
*Observational studies of interventions or non-randomised comparisons in RCTs*							
Craig et al. (2004) Bromet et al. (2005)	Naturalistic 2-year follow-up study investigating antimanic use or non-use in a cohort of first admission BD (with psychotic features). Several baseline and time-varying confounders considered Four-year follow up of the same cohort	155 (2 year follow up) 123 (4 year follow up)	– 29.5 ± 12.1	49.4% 52.8%	Global functioning, time in remission Time to remission and time to relapse	Use of antimanic medications at baseline and 2 years (as well as less use of antipsychotics) associated with higher GAF and greater time in remission Continuously taking antimanic medications or never using medications were associated with shorter time to remission compared to those who started and then discontinued medications	Possible residual confounding by indication
Hafeman et al. (2020)	Long term naturalistic follow up of youth with BD treated with lithium or Other Mood Stabilizers (OMS). Subgroup data extracted for those with ≤ 6 episodes	271 participants contributing to 1850 six-month treatment periods	12.3–12.6 ± 3.2–3.3	50–56%	Suicidality Aggression Functioning	During episodes prescribed lithium there were fewer suicide attempts, and participants had better psychosocial functioning and a lower risk of aggression	Confounding by indication possibly reduced by finding that those prescribed lithium were more unwell at baseline in a sub-proportion
Kessing et al. (2011)	Nationwide registry linkage cohort study comparing first use of lithium vs valproate in BD inpatients	4268	Median 50 years (Quartiles: 39, 60)	58%	Hospital re-admissions	Time to psychiatric hospital admissions was greater for lithium compared to valproate	Possible confounding by indication
Kessing et al. (2012)	Similar registry linkage cohort study to the above study comparing use of lithium vs lamotrigine after first hospitalization for BD	4248	Median 49 years (Quartiles: 48, 60)	59%	Readmissions to hospital, subgrouped by type of bipolar episode; switch to another medication	Switch to another medication, and readmissions were more common in the Lamotrigine group than in the lithium group	Possible confounding by indication
Macneil et al. (2012)	Open label comparative study examining pilot efficacy of CBT vs TAU in a subgroup of those participating in an acute treatment RCT (Conus 2015)	40	21.3–21.8 ± 2.1–2.6	35%	Symptoms, relapse, and functional outcome	Psychotherapy effective in improving functional outcome and reducing depression and overall symptom severity	Potential biases related to selection
Mander (1986)	Registry and medical record-based study of those treated with or without lithium after a first admission for a manic episode	98	35.2–37.5 years ± 13.8–14.3	58.2%	Time to relapse	No group differences for those treated with lithium vs those not treated with lithium. Patients discharged on lithium had longer duration of illness, more severe manic symptoms, and were more likely to be male and from middle class. Lithium treated episodes were associated with significantly greater time to relapse than the episodes where lithium was not used or discontinued	Confounding by indication moderated by the greater severity of symptoms in those prescribed lithium
II. Studies comparing interventions in early vs later course							
*Randomised controlled trials*							
Colom et al. (2010a, b)	Post-hoc exploratory sub-analysis of an RCT comparing those receiving psychoeducation vs non-structured group intervention, divided according to the number of previous episodes	120	Approx. 34 years	63.3%	Time to recurrence and time spent ill	Time to recurrence: Those with 6 or fewer episodes had significantly greater benefit with psychoeducation compared to control group Time spent ill: Those with 6 or fewer (as well as 3 or fewer) episodes and receiving psychoeducation spent less time in any episode polarity compared with controls	Unplanned post-hoc analysis
Ketter et al. (2006)	Post-hoc analysis of RCT investigating the effectiveness of olanzapine vs lithium in preventing recurrence	431	42.3–42.5 ± 12.3–13.1	52–53%	Recurrence to any mood episode, manic episode, depressive episode	Olanzapine resulted in lower recurrence rate compared to lithium in those with 2 or fewer previous episodes. This differential effect was not evident in those with more than 3 prior episodes	Likely adequate power, not clear if hypothesized A priori
Inder et al. (2015)	Post-hoc analysis of RCT examining the efficacy of Interpersonal and Social Rhythm Therapy (IPSRT) compared with Specialist Supportive Care among participants aged 15–36 years	100	26.6 ± 6.0	76%	Cumulative burden of depressive symptoms based on Psychiatric Status Ratings (PSR) from 6–18 months	There was no difference in the outcome between those with 6 or fewer lifetime mood episodes, compared with those with greater lifetime mood episodes	Unplanned post-hoc analysis
Scott et al. (2006)	RCT examining the effectiveness of CBT vs TAU in those with at least 1 episode in previous year	253	39.7–42.7 ± 10.3–11.4	63–67%	Time to recurrence and overall symptom severity	CBT plus treatment as usual (herewith referred to as CBT) was significantly more effective than treatment as usual alone in those with fewer than 6 prior episodes. Within the CBT group, those with less than 6 prior episodes had a lower risk of relapse compared with those with greater prior episodes	A priori planned post-hoc analysis
Swann et al. (1999)	RCT comparing treatment response in acute mania of lithium, divalproex, and placebo with respect to the number of previous episodes of affective disorders	154	39.1–40.4 ± 11.2–12.8 (based on original cohort of 179)	41.9% (for original cohort of 179)	Treatment response (SADS manic syndrome score)	Lithium and divalproex were significantly more effective than placebo for treating acute mania. Those with 5 (or 6) previous episodes did not have a better response with either medication or placebo	Post-hoc analysis describing continuous relationship between number of episodes and treatment response
*Observational studies of interventions or non-randomised comparisons in RCTs*							
Kessing et al. (2014b)	Prospective registry-based linkage study comparing early vs late intervention with lithium in BD	4714	46.7–49.1 Quartiles- 34.2, 59.3	50.4–58.7%	Readmission to hospital following lithium prescription	Patients who purchased lithium after their first contact or first diagnosis had significantly decreased rates of non-response to lithium compared with the rate for patients starting lithium later	Possible confounding by indication
*Pooled analysis of Olanzapine trials comparing early vs late course*							
Berk et al. (2011)	Pooled data from 12 mania, depression and maintenance studies of olanzapine looking at treatment response according to number of episodes				Recurrence and relapse (YMRS score, MADRS, HAMD21 and CGI-BP)	Response rates to olanzapine for mania, depression and maintenance were higher in those with ≤ 5 previous episodes compared to those with > 5 episodes	Pooled post-hoc analyses
Studies included within this analysis	Acute mania studies • Tohen 1999 • Tohen 2000 • Tohen 2002a,b • Tohen 2003 • Perlis 2006 Acute depression studies • Tohen 2003 • Brown 2006 Maintenance studies • Tohen 2003 • Tohen 2004 • Tohen 2005, Ketter 2006 • Brown 2009	1631 1243 1472	40.2 ± 12.3 40.3 ± 11.3 40.5 ± 11.9	62% 53% 56%		Acute mania studies - Better response in YMRS and HAM-D scores for those with 1–5 episodes compared with > 10 lifetime episodes Acute depression studies - Greater response in MADRS scores in those with 1–5 episodes compared with > 10 Maintenance studies - Lower OR and HR with respect to manic relapse after remission in those with 1–5 episodes compared with > 10 lifetime episodes - Lower OR but not HR for depressive relapses after remission in those with 1–5 episodes	

Seven out of 16 RCTs reported results from a priori analyses of primary outcomes, while the others were planned secondary analyses or post-hoc analyses. In terms of study outcomes, manic or depression symptoms were the focus of 10 reports, illness course (including risk of relapse/recurrence or re-hospitalization) in 12 and functioning in three. Eight publications included one or more comparisons between early and later course participants, while the remaining referred to the role of interventions in early course of the disorder. Potential harms were reported in only three publications. There was significant heterogeneity in terms of illness phase (acute vs maintenance), polarity at inclusion, sample age, and outcome measures. For these reasons, a meta-analysis could not be undertaken, and effect size differences could not be estimated.

Tables 2 and 3 describe risk of bias among randomised and non-randomised comparisons, with GRADE assessments provided for clusters of similar interventions. Within early illness course, comparisons that had at least two studies using the same outcome in a similar period as to justify GRADE assessments included those examining the relative efficacy of (i) lithium or quetiapine in acute treatment of mania (ii) lithium or other mood stabilizing agents in preventing recurrence, (iii) mood stabilizers or antipsychotics on functional outcomes in maintenance treatment and (iv) Family Focused Treatment (FFT) vs standard care. In comparisons across early and later illness course, GRADE assessments could only be completed by grouping interventions in higher order categories. These included comparisons of the impact of any pharmacological intervention across early and later course of illness (i) in acute treatment of mania and (ii)in preventing recurrences, as well as (iii) the impact of psychological treatments across course categories in preventing recurrences. The remaining comparisons are described without summative assessments. Additional file 1: Table S1 describes the GRADE assessments. With respect to grouping medications in GRADE assessments or meta-synthesis, we utilized three levels at which medications could be described. First was the level of individual molecules, second was the level of medication class (e.g., mood stabilizer, antipsychotic), and third was an overall grouping combining all medications as ‘pharmacotherapeutic agents’. This was necessary, as not enough studies were available for pair-wise comparison at the level of molecules, or sometimes at the medication class level to draw inferences using GRADE.

**Table 2:** Risk of bias from included randomised controlled trials

Authors	Randomisation process	Deviations from intended interventions	Missing outcome data	Outcome measurement	Selection of reported results	Overall bias
*Comparisons among early course populations*						
Berk et al. (2017)	Low	Low	Some concerns	Low	Some concerns	Some concerns
Conus et al. (2015)	Low	Low	Low	Low	Low	Low
Detke et al. (2015)	Low	Low	Low	Low	Low	Low
Kessing et al. (2013)	Low	Low	Low	Low	Low	Low
Kessing et al. (2014a)	Some concerns	Low	low	low	Some concerns	Some concerns
Miklowitz et al. (2003)	Low	Low	Some concerns	Low	Low	Some concerns
Miklowitz et al. (2008)	Low	Low	Low	Low	Low	Low
Miklowitz et al. (2014)	Low	Low	Low	Low	Low	Low
Patino et al. (2021)	Low	Low	Some concerns	Low	Low	Some concerns
Perry et al. (1999)	Low	Low	Low	Low	Low	Low
Strakowski et al. (2016)	High	Low	High	High	High	High
*Comparisons across early vs later course of illness*						
Colom et al. (2010a,b)	Some concerns	Low	Low	Low	High	High
Ketter (2006)	Low	Low	Low	Low	High	High
Inder et al. (2015)	Low	Low	Low	Low	High	High
Scott et al. (2006)	Low	Low	Low	Low	Some concerns	Some concerns
Swann et al. (1999)	Some concerns	Low	Low	Some concerns	High	High

**Table 3:** Risk of bias from included non-randomised studies

Study	Sub-group analysis or outcome	Bias due to confounding	Bias in selection of participants	Bias in classification of interventions	Bias due to deviations from intended interventions	Bias due to missing data	Bias in outcome measure-ment	Bias in selection of reported results	Overall bias
*Comparisons in early course populations*									
Bromet et al. (2005)	–	Serious	Low	Moderate	Serious	Low	Low	Serious	Serious
Craig et al. (2004)	–	Serious	Low	Moderate	Serious	Low	Low	Serious	Serious
Hafeman et al. (2020)	–	Moderate	Moderate	Low	Moderate	Moderate	Low	Serious	Serious
Kessing et al. (2011)	–	Moderate	Low	Low	Low	Low	Low	Low	Moderate
Kessing et al. (2012)	–	Moderate	Low	Low	Low	Low	Low	Low	Moderate
MacNeil (2021)	–	Serious	Moderate	Moderate	Serious	Low	Low	Serious	Serious
Mander (1986)									
	Treatment episode	Serious	Serious	Low	Moderate	Low	Low	Moderate	Serious
	Persons prescribed lithium	Serious	Low	Low	Low	Low	Low	Moderate	Serious
*Comparisons of early vs late course of illness*									
Kessing et al. (2014b)	–	Moderate	Low	Low	Low	Low	Low	Low	Moderate
Berk et al. (2011)									
	Mania remission acute mania studies	Moderate	Low	Moderate	Low	Moderate	Low	Moderate	Moderate
	Mania remission maintenance studies	Moderate	Low	Moderate	Low	Low	Low	Moderate	Moderate
	Mania relapse	Moderate	Moderate	Moderate	Low	Low	Low	Moderate	Moderate
	Depression remission acute depression studies	Moderate	Low	Moderate	Low	Moderate	Low	Moderate	Moderate
	Depression remission maintenance studies	Moderate	Low	Moderate	Low	Low	Low	Moderate	Moderate
	Depression relapse	Moderate	Moderate	Moderate	Low	Low	Low	Moderate	Moderate

Results are described according to several levels, corresponding to the Participant, Intervention, Comparison, Outcomes (PICO) framework utilized in our selection criteria. The descriptions correspond to (i) type of comparison (which were also our main objectives), (ii) type of intervention (iii) phase of illness (sub-population) and (iv) outcomes of interest.

### Intervention outcomes within early course studies

As outlined in Table 1, the studies meeting our criteria for early course of illness varied widely with respect to mean or median sample age. Six studies referred to data from adolescents (between 12 and 19 years of age), four studies included young adults (mean age between 20 and 35 years) and six studies included adults with a mean or median age of 35 and over.

### Pharmacotherapy in early illness course

#### Acute treatment of mania in early illness course

##### Lithium vs quetiapine

In a six-week double blind RCT, 109 adolescents were included for their first hospitalisation for a manic or mixed episode (Patino et al. 2021). Investigators compared use of quetiapine (400 to 600 mg) and lithium (1.0 to 1.2 mEq/L) and observed a greater reduction in manic symptoms with quetiapine than lithium (mean difference = 2.2 points, *p* < 0.001) and a higher response rate (72.4% vs 49%; OR 2.73, 95%CI 1.23–6.05). Emesis (26%) was common in the lithium group, reflecting relatively high target serum lithium levels (1.0–1.2 mmol/L). Sedation was more common in the quetiapine group (63.8% vs 28%; OR 4.7, 95% CI 2.1–10.5). Those in the quetiapine group had more weight gain (+ 3.7 kg vs + 1.3 kg, *p* = 0.02) than the lithium group. Side effects common among both groups included headaches (55% vs 61%, respectively), tremors (36% vs 28%), and nausea (31% vs 39%).

In a second study that focused on changes in brain activation among 42 adolescents during treatment with either lithium or quetiapine, response rates were reported as secondary outcomes (Strakowski et al. 2016). There was no significant difference across groups (estimated OR 1.79, 95% CI 0.52–6.1).

*GRADE assessment*: Considering these two studies, no conclusions could be drawn about the relative efficacy of these medications in treating acute mania in the early course of illness (Additional file 1: Table S1).

##### Olanzapine vs chlorpromazine

In a double blind 8-week RCT (Conus et al. 2015), the efficacy of olanzapine plus lithium in treating severe first-episode psychotic mania was compared with that of chlorpromazine plus lithium. There were no significant group differences with respect to remission (OR 1.4, 95%CI 0.51–3.8) or response (OR 1.09, 95%CI 0.37–3.22). Although adverse events were not significantly different across groups, more than half of all participants experienced moderate to severe sedation, nearly a third experienced significant weight gain and over one-fifth experienced concentration difficulties, tiredness, and dry mouth. Risk of bias was judged to be low.

##### Mood stabilizers, antipsychotic or antidepressant medications- comparing continued use, discontinuation and never starting medications

The role of compliance with medications was examined in a naturalistic cohort study of first episode psychosis participants, where results were reported separately for 123 participants seeking help for manic or depressive episodes of BD (Bromet et al. 2005). Over 4 years of follow-up, the sub-group of participants who did not receive medications were as likely to remit as those who continuously took medications. Discontinuous use of medications was associated with a lower likelihood of remission compared to not taking medications (OR 0.20, 95%CI 0.08–0.51 for antimanic medications). It should be noted that this finding was biased due to residual confounding by indication.

#### Acute treatment of depression in the early illness course

##### Olanzapine-fluoxetine combination vs placebo

In an RCT among adolescents (Detke et al. 2015), the investigators examined safety and efficacy of an olanzapine/fluoxetine combination (OFC) for the acute treatment of bipolar depression. The sample had a median of one past manic episode and two past depressive episodes, indicating an early course of illness. The mean change from baseline to week 8 on the Children’s Depression Rating Scale- Revised total score was significantly greater for the OFC group than for the placebo group (mean difference − 5.0, 95% CI − 8.3: − 1.8), along with significantly better outcomes on a range of secondary outcomes. The most frequent adverse events in the OFC group were weight gain (20% OFC vs 1.2% placebo), increased appetite (16.5%), and somnolence (16%). Treatment-emergent hypertriglyceridemia (7.1%), increases in prolactin (58%), and corrected QT interval (≥30 ms, 12%) were also common in the OFC group. This RCT was associated with a low risk of bias.

#### Prevention of recurrences in early illness course

##### Lithium vs quetiapine

Berk and colleagues (2017) conducted an RCT comparing these agents as maintenance treatments in first-episode psychotic mania. Although symptomatic outcomes were secondary, lithium was superior to quetiapine with respect to global illness severity, depressive symptoms and functioning over a 1-year follow-up period. The quetiapine group worsened while the lithium group showed mild improvement: CGI BP change for quetiapine was -1.7 (0.4), and that for lithium was 0.7 (0.4). Odds of remaining in remission at 12 months based on CGI-BP overall severity scores were higher with lithium than quetiapine (OR 17.9, 95%CI 2.7–116.9).

##### Lithium vs valproate

In a registry-based comparative study by Kessing et al. (2011), the authors examined rate of psychiatric admissions for 4268 participants receiving lithium vs valproate for a first hospital admission for BD. After adjusting for baseline demographic features, treatment history and taking some comorbid disorders into account, treatment with valproate resulted in significantly more hospital admissions compared to lithium (HR = 1.33, 95% CI 1.18–1.48).

##### Lithium vs lamotrigine

A similar approach (as in Sect. 3.2.2) was utilized to examine the relative efficacy of lithium and lamotrigine using a registry-linkage approach (Kessing et al. 2012). In this sample (N = 4248), risk of rehospitalization or switch to another medication was examined adjusting for baseline and time-varying confounders. The rate of hospitalization for depression was significantly higher in the lamotrigine group over follow-up (HR 1.52, 95%CI 1.27–1.81), an effect that was more pronounced if the index episode was manic (HR 2.08, 95% CI 1.38–3.14). Lamotrigine was also associated with a higher risk of medication change or augmentation irrespective of index episode polarity.

##### Lithium vs olanzapine

In a post-hoc analysis from a 12-month continuation phase trial (Ketter et al. 2006), recurrence risk with olanzapine was compared to that with lithium in BD I patients with two or fewer episodes. Treatments were similar in their efficacy in preventing recurrence to any mood episode, but olanzapine was associated with a significantly lower risk of recurrence to mania (OR 0.06, 95%CI 0.01–0.47). This differential effect was not apparent in risk of recurrence of depression or in subgroups with three or more episodes. This study suffered from a high risk of bias due to lack of consideration of confounders within several post-hoc comparisons reported.

##### Lithium vs other agents

The relative effectiveness of lithium was explored in two naturalistic comparisons from a file audit registry based study (Mander 1986). The authors explored the relationship between being prescribed lithium and the probability of remaining well after a first admission for acute mania in two analyses, (a) among participants and (b) among episodes of lithium treatment. The first comparison included all those who were either prescribed or not prescribed lithium at index episode. In the second comparison, those discontinuing lithium were reclassified as ‘not on lithium treatment’, contributing to episodes where participants were either on lithium treatment or not. While being prescribed lithium in the first comparison was not associated with a lower likelihood of a recurrence (HR 1.04, 95%CI 0.60–1.79), being compliant with lithium in the second comparison did (HR 0.34, 95%CI 0.20–0.59). The direction of bias due to confounding by indication could not be fully ascertained.

*GRADE assessment*: Considering these three observational studies and two RCTs, it is likely that lithium may be more effective than other mood stabilizing agents in preventing recurrences of any mood episode in early illness course. However, there were contrary findings with respect to olanzapine for preventing manic episodes. The quality of this evidence is low given the risk of bias among included studies and the possibility of publication bias (Additional file 1: Table S1). These differences appear unrelated to study design (RCT vs non-randomised comparison), the gender distribution of the included studies (22–59%) and the mean or median sample age (12–50 years).

### Impact of pharmacotherapy on functioning in the early illness course

#### Antipsychotics vs mood stabilizers

##### Regularity of antipsychotic and mood stabilizing medication use

The differential impact of these medications on functioning was examined in the naturalistic cohort study mentioned previously (Bromet et al. 2005). In the first 2 years of follow-up (Craig et al. 2004), higher Global Assessment of Functioning score (GAF > 70) was associated with regular use (> 75% of the time) of mood stabilizing medications compared with less regular use. This was evident regardless of whether use occurred early (OR 5.96, 95%CI 2.04–17.40) or later in the episode of care (OR 3.51, 95%CI 1.12–11.0). In contrast, regular use of antipsychotic medications early in the episode of care was associated with lower global functioning (OR 0.20, 95%CI 0.04–0.91).

##### Lithium vs quetiapine

In the 12-month follow-up study described previously (Berk et al. 2017), lithium treatment was associated with an improved GAF score from baseline (mean change − 7.9, SD 4.0) while those on quetiapine worsened with respect to their global functioning (mean change 11.7, SD 4.2).

*GRADE assessment*: Based on these two studies of patients in the early course of BD, mood stabilizers may be associated with better global functioning over 12–24 months of follow up, compared to the use of antipsychotics. This evidence is of very low certainty given the high risk of bias, imprecision, indirectness, relatively few studies, and the possibility of publication bias (Additional file 1: Table S1).

#### Lithium vs other agents

In a sample of youth with BD I, II or NOS prescribed lithium or other agents (Hafeman et al. 2020), the authors provided subgroup data for those participants with 6 or fewer lifetime mood episodes. Units of analyses were 6-month treatment periods when participants were treated with lithium, or with other agents. Periods of lithium treatment were associated with better psychosocial functioning based on the participants’ worst score on the Longitudinal Interval Follow-up Evaluation (LIFE) Psychosocial Functioning tool (PSF, *β* = − 0.46, 95%CI − 0.90 to − 0.03) during 6-month treatment periods and the analysis accounted for demographic and clinical confounders. The LIFE Psychosocial Functioning tool assesses functional domains such as work/school, interpersonal, recreation and satisfaction domains.

### Psychological treatments in early illness course

All studies identified utilized adjunctive psychological interventions alongside standardized or routinely available pharmacotherapy or other treatment as usual delivered across both the intervention and comparison arms. Interventions were also delivered in the maintenance phase with 1- to 2-year follow-up periods.

#### Cognitive behavioral therapy

In a sub-cohort of those recruited for an aforementioned trial in first-episode psychotic mania (Conus et al. 2015), recovery-oriented CBT was offered to a sub-group of participants (Macneil et al. 2012) and outcomes were examined at 18-months. Outcomes of those who received the intervention were compared with those of an individually matched group who received fewer than 4 intervention sessions or did not continue with the intervention. Recovery-oriented CBT was associated with lower depression symptom severity at follow up (end point group mean difference on Hamilton Depression Rating Scale = 4.0, 95% CI 1.6–6.4). The intervention group also reported better functioning on the Social and Occupational Functioning Assessment Scale (mean difference = 15.1, 95% CI 6.0–24.2). However, risk of bias was judged to be high, related to confounding, deviations from intended interventions, and selection of reported results.

Further, in a large RCT of CBT for preventing recurrences in those with established BD (Scott et al. 2006), data pertaining to those with less than six prior episodes were extracted. CBT plus treatment as usual (TAU) was significantly more effective than treatment as usual alone in those with fewer than six prior episodes. Median time to any recurrence was 64 weeks in the CBT group compared with 33 weeks in the TAU alone group.

#### Family focused therapy (FFT)

The impact of adjunctive FFT alongside psychopharmacological treatment was investigated in three RCTs of participants predominantly in early illness course. Comparators included enhanced care, crisis management or briefer family interventions. While time to recurrence was longer with FFT (73.5 weeks ± 28.8 vs 53.2 weeks ± 39.6) in one trial (Miklowitz et al. 2003), this was not different between groups in the other two trials focused on adolescents. In the latter cohorts, FFT was associated with improvements on secondary outcomes, including time spent in depressive episodes in one trial (Miklowitz et al. 2008) and severity of manic symptoms in the other (Miklowitz et al. 2014).

*GRADE assessment*: Considering these three studies, firm conclusions could not be drawn about the relative efficacy of FFT in preventing recurrence of any mood episode in early course participants (Additional file 1: Table S1).

### Multi-component interventions in early illness course

#### Specialized outpatient care

The role of specialized care for mood disorders was examined in an RCT by Kessing et al., who enrolled 158 patients discharged after their first, second or third hospital admission for BD (Kessing et al. 2013). Care in the specialized mood disorder clinic included guideline concordant pharmacological interventions and group-based psychoeducation, whereas standard care included routine outpatient mental health services. The latter could be variable and include general practitioners, outpatient psychiatrists or community mental health services. Risk of subsequent readmission was significantly lower in those treated in the specialized mood disorder clinic (HR = 0.60, 95%CI 0.37–0.97) and these participants had greater satisfaction with care compared to those in standard care. Those receiving specialized care were more likely to receive a mood stabilizer or an antipsychotic. Risk of bias was deemed to be low. Although not statistically significant, differences between groups were more prominent in a smaller subgroup of those under age 26 (HR 0.33, 95%CI 0.10–1.07; *p* = 0.064), favoring early treatment in the specialized mood disorder clinic (Kessing et al. 2014a).

### Comparing outcomes across early vs later course of illness

Among the included studies, we compared outcomes across early and later illness course using subgroup data from those studies that included populations with varying number of episodes at baseline.

### Pharmacotherapy in early vs later illness course

Pharmacological treatments included in studies comparing those in early vs later course were lithium, valproate/divalproex, lamotrigine, and olanzapine.

#### Treatment of acute mania in early vs later illness course

##### Olanzapine, mood stabilizers or placebo

Several olanzapine trials that aimed to treat acute mania, acute depression, and to prevent recurrences examined the role of number of previous episodes in treatment efficacy. Findings related to treatment of acute mania, acute depression and prevention of recurrences have been summarized in a pooled re-analysis (Berk et al. 2011) that included data from 12 RCTs.. After adjusting for baseline demographic, clinical and treatment characteristics, response rates in treatment studies of acute mania and stabilization phase of maintenance studies were significantly higher for patients with 1–5 prior episodes compared to those with > 10 prior episodes on the Young Mania Rating Scale (YMRS, OR 1.5, 95%CI 1.1–2.0) and on CGI-BP (OR 2.2, 95%CI 1.6- 3.0).

##### Lithium, valproate/divalproex, and placebo

In an RCT (Swann et al. 1999) comparing efficacy of lithium, valproate, and placebo in treating acute mania, post-hoc analyses explored efficacy of treatments against number of previous episodes. In this analysis, those with fewer than six previous episodes had no difference in treatment response to those with more episodes, although risk of bias was high. Tolerability data were not reported across early and later course.

*GRADE assessment*: Considering the above two studies, no conclusions could be drawn about whether pharmacological interventions are more effective in treating acute mania in the early course of illness compared to later illness course (Additional file 1: Table S1).

#### Treatment of acute depression in early vs later illness course

##### Olanzapine, mood stabilizers or placebo

In the pooled analysis of olanzapine studies described above (Berk et al. 2011), response rates for depression studies were significantly higher for patients with 1–5 episodes compared to those with > 10 prior episodes on the Montgomery Åsberg Depression Rating Scale (OR 1.6, 95%CI 1.02–2.4), but not on CGI-BP (OR 1.3, 95% CI 0.9–2.0).

#### Prevention of recurrences in early vs later illness course

##### Olanzapine, mood stabilizers, or placebo

Among trials using olanzapine that aimed at preventing recurrences (Berk et al. 2011), hazard ratios for manic recurrences were significantly lower for those with 1–5 prior episodes (HR 0.5, 95%CI 0.3–0.8) compared with those with > 10 previous episodes. However, risk of recurrence to depression was not significantly different across groups (HR 0.7, 95%CI 0.4–1.2).

##### Lithium

In a registry-based observational study over a 16 year-follow-up period, Kessing and colleagues (2014b) compared risk of rehospitalization after commencing lithium among patients who started treatment early or later in illness course. The authors defined early or late introduction of treatment in one of two ways, (i) treatment introduced following a first contact or after later contacts and (ii) treatment following a single manic/mixed episode or after diagnosis of recurrent BD. Regardless of the definition used, risk of rehospitalization was significantly lower in patients who started lithium early compared to patients who started lithium later (HR = 0.87, 95%CI 0.76–0.91 and HR = 0.75, 95%CI 0.67–0.84 respectively).

*GRADE assessment*: Based on the two studies reported above, there is modest evidence that pharmacological interventions may be more efficacious in preventing mood recurrences or rehospitalisations in earlier rather than later course of illness. As outlined in Additional file 1: Table S1, certainty of this evidence is low given the relative paucity of studies, moderate risk of bias, indirectness of evidence, and possible publication bias.

### Psychological interventions in early vs later illness course

In these comparisons, all studies included utilized adjunctive psychological treatments delivered with the aim of preventing recurrences.

#### Cognitive behavioral therapy

In the aforementioned RCT of CBT in those with BD, Scott and colleagues (2006) conducted a planned secondary analysis based on the number of prior mood episodes. Within the CBT group, if pattern of recurrences in the subgroup with < 6 episodes are compared with three other subgroups (classified according to 6–11, 12–29, and ≥ 30 prior episodes), the adjusted HR for recurrence in those with 6–12 episodes was 3.01 (95% CI 1.07–8.44), with 12–29 past episodes was 3.89 (95%CI 1.48–10.24), and with 30 or more episodes was 5.33 (95%CI 2.03–14.02). In those who received TAU alone, there was an increase in the adjusted HR for recurrence in the three subgroups with more prior episodes compared with < 6 episodes, but the overall change was less clear cut (e.g., for 30 + episodes versus < 6 episodes: adjusted HR 1.86, 95%CI 0.85–4.06).

#### Psychoeducation

Similar post-hoc exploratory analyses were conducted in an RCT comparing participants who received structured group psychoeducation compared with a supportive group intervention (Colom et al. 2010a). Psychoeducation significantly improved time to recurrence for participants with ≤ 6 previous episodes (log-rank 4.3, *p* = 0.04), but not for those with > 6 prior episodes. Additionally, following psychoeducation, patients with ≤ 6 episodes showed reduction in time spent acutely ill in any episode polarity, whereas patients with > 14 episodes did not benefit.

#### Interpersonal and social rhythms therapy (IPSRT)

Finally, in an RCT examining the relative efficacy of IPSRT vs Specialized Supportive Care (SSC (Inder et al. 2015)), the authors provided data on the relative efficacy of IPSRT and SSC among persons considered to be in early vs later illness course. The primary outcome was cumulative burden of depressive symptoms in study weeks 26–78, or for 1 year after the intervention. In this post-hoc analysis, those with ≤ 6 lifetime episodes did not differ from those with > 6 episodes among those receiving either IPSRT or SSC.

*GRADE assessment*: Based on these three studies, no firm conclusions could be drawn regarding whether psychological interventions are more efficacious in prevention of recurrence in earlier vs later course of illness (Additional file 1: Table S1).

## Discussion

We used systematic methodology to review the effects of pharmacological and adjunctive psychological interventions on symptomatic, course and functional outcomes in populations in the early course of threshold BD I or II. We identified that lithium treatment may be associated with a lower risk of recurrence of mood episodes compared with several other agents among those in the early course of illness. In this population, adherence to antipsychotic agents was also associated with worse psychosocial functioning over the first one or two years of follow-up when compared to those who were not compliant with antipsychotic treatment and to those on lithium. Firm conclusions could not be drawn about psychological interventions due to variable outcomes and comparisons among the included studies. However, there were promising findings supporting CBT and FFT in participants in early illness course. When comparing intervention effects across early and later course of illness, there was some evidence that pharmacological interventions were more likely to be effective if used earlier in the illness course.

Within the first few episodes after illness onset, there was evidence of efficacy for several interventions including mood stabilizers, antipsychotics, and psychological interventions across a range of outcomes. The most consistent higher order finding was the relative effectiveness of lithium over other agents in preventing recurrence to any polarity of illness. This has also been identified in unselected samples of adults with BD (Severus et al. 2014). However, one of the included studies suggested that olanzapine and lithium were similar in their efficacy in preventing recurrences to any polarity in early illness course (Ketter et al. 2006), and olanzapine was more effective in preventing manic episodes. This parallels the finding from one of the RCTs in which quetiapine was more effective than lithium in treating acute episodes of mania (Patino et al. 2021). Thus, antipsychotics may have a greater effect on manic episodes in early illness course, which is also supported by data on those with BD in general (Carvalho et al. 2014). Three studies also indicated that antipsychotics may be associated with significant side effects when used in the treatment of acute mood episodes in early illness course, in comparison with other agents. This raises questions regarding the risk–benefit balance of antipsychotics in acute and continuation treatment. Such concerns were reflected in an RCT comparing antipsychotic treatments for varying durations after an acute manic episode (Yatham et al. 2016). Continuation of these medications beyond 6 months was associated with a higher risk of adverse events without clear benefit in preventing recurrences. Our findings also suggest the possibility that continued use of antipsychotics may be associated with worse psychosocial functioning over 1–2 years when utilized for preventing recurrence in early illness course. However, this finding should be interpreted with caution given the different comparisons included in this observation, and the heterogeneity amongst antipsychotics in terms of their pharmacodynamic effects and side effect profiles (Jauhar and Young 2019). In all, there may be value in considering mood stabilizers, primarily lithium for maintenance treatment in early illness course over antipsychotic medications, while antipsychotics may have a role in acute treatment. Shared decision-making involving patients and caregivers, weighing the risks and benefits of interventions in different phases of illness can help navigate treatment decisions.

We also identified single studies with low risk of bias in early course participants that indicated efficacy of an olanzapine-fluoxetine combination (Detke et al. 2015) in treating acute depression and psychoeducation in preventing recurrences (Perry et al. 1999) in early illness course. Finally, there was evidence that combining guideline concordant pharmacotherapy and group-based psychoeducation in a specialized mood disorder service (Kessing et al. 2013) was more effective than standard care in preventing recurrences and improving patient satisfaction. This study parallels other findings from our review in highlighting the benefits of tailored pharmacotherapy when combined with group-based psychoeducation in early illness course. Given that such patients are likely to be adapting to their relatively recent diagnoses, psychoeducation interventions may improve adherence, and therefore treatment effectiveness.

Regarding our a priori secondary objective to compare response to the same treatment across early and later course of illness, there were fewer consistent findings. There was evidence from a pooled re-analysis of RCTs (Berk et al. 2011) and an observational study (Kessing et al. 2014b) that pharmacological interventions were more effective, either when utilized in the first hospitalized episode or within the first five episodes after onset. Despite the moderate risk of bias, including the possibility of publication bias, this preliminary finding points to the possibility of illness progression or ‘neuroprogression’ (Kupka et al. 2021) amongst at least a subgroup of participants. It is hypothesized that in this subgroup, recurrent illness is associated with deteriorating functioning and treatment response, perhaps due to a progressive pathophysiological process driven by the primary illness or secondary impacts of treatment or comorbidity (Berk et al. 2009). However, there is insufficient direct evidence for this hypothesis. Alternatively, some participants with a more severe baseline illness might develop more episodes before they access treatment, and because of underlying prognostic factors, do poorly with treatment. Although this has been controlled to some degree by adjusting for baseline sociodemographic factors, illness severity, and prior hospitalizations, there may be residual confounding. It is notable that other systematic reviews (Bratti et al. 2003; Hui et al. 2019) have also highlighted an unclear relationship between response to treatments and the number of prior episodes.

We could not identify a consistent finding regarding differential response to psychological treatments in early vs. later illness course. While two psychological intervention trials supported the possibility of better treatment response in earlier illness course (Scott et al. 2006; Colom et al. 2010b), one did not (Inder et al. 2015). When a broader number of prior episodes was considered in the Systematic Treatment Enhancement Program for Bipolar Disorder (STEP-BD) study (Peters et al. 2014), participants were more likely to recover with any psychological intervention if they had fewer episodes. However, there were differential patterns of recovery depending on the type of psychological intervention and the number of prior episodes. Finally, the lack of consistent findings with regards to psychological intervention trials may also be due to heterogeneity related to interventions, comparisons, and outcomes rather than an absence of evidence regarding psychological interventions in early illness course.

Finally, interventions in early course of BD could be understood within a broader transdiagnostic context, given the overlap and commonalities between BD, recurrent major depression and non-affective psychoses, especially in the early course of these disorders (Caspi et al. 2020; Neumann et al. 2016). Given that depression is the most common onset polarity in BD, early intervention for BD is likely to closely parallel, or complement intervention efforts in the early course of depressive disorders. It is also pertinent that pre-onset interventions for BD frequently target depression, subthreshold mood symptoms, anxiety and other high-risk states for BD (Saraf et al. 2021). The interventions utilized in such high-risk states were similar to those we identified to have evidence of efficacy in early post-onset illness course. Similarly, psychosis and BD type I also share similarities in their age of onset (Lin et al. 2006; Liu et al. 2013). Several studies included in our review (Conus et al. 2015; Bromet et al. 2005; Berk et al. 2017) utilized cohorts with first episode psychosis including first episode psychotic mania, and non-affective psychoses. In these populations, the efficacy and tolerability of interventions for non-affective psychosis and mania may have similarities. However, the relatively limited intervention research in the early course of BD compared to that of schizophrenia or psychoses more broadly (Correll et al. 2018) highlights the need for comparative effectiveness trials in the former population.

### Limitations

This review is characterized by our multipronged definition of early illness course, which was chosen as a pragmatic strategy so that results can guide treatment. While this could have led to inclusion of heterogeneous populations, making interpretation of findings difficult, the broad definition allowed us to canvas a wide range of studies and collate evidence in early illness course. Further, we limited the definition of early course to number of episodes, rather than time elapsed from illness onset to limit heterogeneity. There may also have been measurement error in defining course using number of episodes, particularly given recall effects, which may be more prominent for prior depressive or mild hypomanic episodes (Tremain et al. 2020). Our findings are also limited by the quality of primary studies, a majority of which suffered from a moderate to high risk of bias as well as the possibility of publication bias. Finally, our comparison of studies across early and later illness course was limited by our search, which was not designed to capture all studies in the later illness course.

## Conclusions and taskforce recommendations

1. More data are needed on the impact of pharmacological, psychological, and other interventions in early illness course. Our review indicates the need for high quality RCTs in this population, with a focus on symptomatic, functional, and quality of life outcomes. Combinations of psychological and pharmacological interventions may have synergistic benefits, although optimal interventions or the possible combinations of interventions that may be the most effective for this early course group are not yet clear.
2. Tolerability (and acceptability) of interventions needs closer attention in both pharmacological and psychological treatment trials, particularly in the maintenance phase. Large effectiveness trials with pragmatic outcomes (e.g., time to all-cause discontinuation or quality of life) in naturalistic settings can also help to better understand the risk–benefit balance. Establishing registries or collating naturalistic treatment data from several centers could also help improve our understanding of tolerability, particularly rarer adverse events, or longer-term risks.
3. Recruiting large numbers of early course participants in clinical trials or naturalistic studies will likely require multi-center approaches. In addition, psychoeducation interventions may be needed early in the seeking help process, as many participants may not otherwise be motivated to seek care or continue with care in the early illness course. Education interventions for caregivers, families and primary care providers could also support early help-seeking and appropriate referrals (Berk et al. 2013). Future studies should include a broad range of youth and adults judged to be in the early course of illness.
4. A definitive head-to-head, multi-center RCT is necessary to compare the effectiveness of lithium against other mood stabilizers and/or antipsychotics in participants in the early course of BD. Outcomes should be determined in the medium term (1–2 years) and should include recurrence risk, functioning and tolerability. Adjunctive psychological treatments should be provided to participants and controlled for in analyses.
5. With respect to psychological therapies, there is a need to identify the relative benefits of FFT, psychoeducation, CBT and IPSRT in the early course of BD. Such a study could explore whether individually delivered and group-based interventions could complement each other when delivered in combination(s). Alternatively, the relative benefits of such interventions could be directly compared. Given that all psychological intervention studies included in our review focused on preventing recurrences, there is a need for greater focus on treating acute episode of illness, particularly depression, where there are fewer effective treatment options.
6. Given the promising role of lithium in preventing recurrences in early illness course, as well as the evidence for combining pharmacotherapy with psychoeducation (Kessing et al. 2013), a combination of lithium and psychoeducation interventions should be evaluated in early illness course. Understanding patterns of treatment discontinuation in naturalistic intervention cohorts receiving such a combination could also help understand the risks and benefits of lithium in this population.
7. While evidence comparing early vs later course of illness is post-hoc, it is neither feasible nor ethical to randomize participants to receive or not receive specific interventions depending on their course of illness. However, among those considered to be in early illness course, Sequential Multiple Assignment Randomised Trials (Murphy 2005) could identify the role of specific interventions while balancing efficacy and tolerability. For example, in such a trial, participants in early illness course could receive psychological interventions or lithium early while antipsychotics or anticonvulsants are offered to those who find these first line agents ineffective. Future consensus-based approaches could also help identify the optimal assignment steps in such trials.
8. We conceptualized the early illness course across BD I and II to include those having experienced up to six lifetime mood episodes, and in BD I to include those in the first treatment seeking episodes of mania or having up to three lifetime manic episodes. This may help define the early-stage concept for BD in staging nomenclature (Kupka et al. 2021), with further clarifications including time-elapsed from diagnosis, functioning, and inter-episodic symptoms. This could be refined further in future consensus-based studies. However, in the absence of a clear threshold at which treatment response changes or other markers differ across groups, early course or stage could also be considered a continuum from pre-onset symptoms to subthreshold mood episodes and the first few threshold mood episodes. The interaction of life course with illness course also merits consideration in future studies and in the conceptualization of stages (Bolton et al. 2021).
9. Assessing the number of lifetime mood episodes to define early illness course may require the use of structured instruments (Tremain et al. 2020). For example, the National Institute of Mental Health Life Chart Method (Leverich and Post 1996) can be utilized for retrospective monthly ratings of mood and functioning to ascertain the existence of clear mood episodes. The Affective Disorders Evaluation (Sachs et al. 2003) may be a less cumbersome instrument, with ordinal response categories better suited for earlier course of illness.
10. In future studies comparing those in early and later illness course or stage, a wider set of baseline confounders should be considered, particularly the presence of comorbid developmental, anxiety, substance use, and personality disorders. Longer-term observational studies of individuals could partly address the confounding by indication that occurs in group-level analyses. The same individuals’ treatment response in early course could be compared with response in later illness course, possibly in registry-based studies. However, initial treatment response should be accounted for in such analyses, as this may affect treatment choices later in illness course.
11. Given the evidence for pharmacological and psychological interventions, those with BD in their early illness course should be offered access to safe and effective interventions. The best models to implement such interventions need further research, often in local health systems. Ethical concerns regarding early intervention could be balanced with patient preference in shared decision-making paradigms. In all, early intervention for BD should also include those in the early course of syndromal BD I or II alongside interventions in the pre-onset phase.

## Supplementary Information

**Additional file 1: Table S1.** GRADE assessments. **Table S2.** PRISMA (2020) Checklist.

## Data Availability

The datasets used and/or analysed during the current study are available from the corresponding author on reasonable request.
